# Preoperative quadriceps muscle strength deficit severity predicts knee function one year after anterior cruciate ligament reconstruction

**DOI:** 10.1038/s41598-022-09816-3

**Published:** 2022-04-06

**Authors:** Do Kyung Kim, Geon Park, Joon Ho Wang, Liang-Tseng Kuo, Won Hah Park

**Affiliations:** 1 Department of Sports Medicine Center, Samsung Medical Center, School of Medicine, Sungkyunkwan University, 81 Irwon-ro, Gangnam-gu, Seoul, 135-710 South Korea; 2 Department of Orthopaedic Surgery, Samsung Medical Center, School of Medicine, Sungkyunkwan University, Seoul, Korea; 3 Department of Orthopaedic Surgery, Sports Medicine Center, Chang Gung Memorial Hospital, No. 6 West Sec, Chia-Pu Road, Putz City, Chiayi, 613 Taiwan; 4 Department of Medicine, Chang Gung University, Taoyüan, Taiwan

**Keywords:** Health care, Medical research, Rheumatology

## Abstract

Quadriceps strength is critical for patients with anterior cruciate ligament (ACL) reconstruction; however, little is known about the relationship between preoperative quadriceps strength deficit and postoperative subjective knee functions. The study aimed to investigate the relationship between preoperative quadriceps strength and postoperative knee function in patients after ACL reconstruction. Seventy-five male patients with primary ACL reconstruction surgery with hamstring autografts between 2014 and 2017 were included. An isokinetic dynamometer assessed quadriceps strength while self-reported knee functions were measured by the International Knee Documentation Committee (IKDC) and Lysholm scores at baseline and 1 year after surgery. The three identified groups (Q1–Q3) were classified according to the preoperative quadriceps muscle strength deficit. Q1 were patients with < 25% quadriceps muscle strength deficit, Q2 showed a 25–45% deficit, and Q3 included those with a deficit > 45%. We compared knee functions between the three groups and examined the associations between preoperative variables and functional knee outcomes. The preoperative quadriceps muscle strength deficit had a negative association with the knee functional scores at 1 year follow-up including the IKDC score (r_s_ = − 0.397, *p* = 0.005) and the Lysholm score (r_s_ = − 0.454, *p* < 0.001), but not other factors. Furthermore, only the Q1 group, with < 25% deficit in preoperative quadriceps muscle strength, showed a significant correlation in postoperative IKDC score (*r* = − 0.462, *p* = 0.030), and Lysholm score (*r* = − 0.446, *p* = 0.038). Preoperative quadriceps muscle strength deficit had a significant negative relationship with postoperative function at 1 year following ACL reconstruction.

## Introduction

Anterior cruciate ligament (ACL) reconstruction surgery is a standard procedure for treating ACL injury. The goals of ACL reconstruction are to restore normal knee biomechanics and laxity, to help patients recover to pre-injury activity levels, and to improve the quality of daily life^1,2^. However, many patients still experience muscle weakness and fail to return to pre-injury activity level, even after successful surgery and rehabilitation^3^.

Adequate quadriceps strength, functional outcomes, and psychological preparedness are standard criteria for returning to sports^4^. Quadriceps weakness is critically detrimental to the return to sports because it is significantly associated with poor functions and deteriorated quality of life after ACL reconstruction^5–7^. Furthermore, patients with an asymmetry of quadriceps strength are also at higher risk of re-injury after ACL reconstruction than the normal population^8,9^. Although quadriceps muscle strength deficit is expected^10–12^ and essential for ACL reconstruction outcomes, it is not convenient to regularly assess the isokinetic muscular strength during follow-up due to financial and time costs. Instead, self-reported questionnaires evaluate the knee functional status by identifying pain, functional problems, and physical activity levels. The Lysholm score, International Knee Documentation Committee form (IKDC), Hospital for Special Surgery score, and Knee Outcome Survey score are popular for qualitatively assessing subjective knee functions after ACL reconstruction^13,14^.

Preoperative quadriceps strength has been proposed as a predictor for ACL reconstruction functional outcomes measured^15,16^. Severe preoperative quadriceps strength deficit is a factor for persistent quadriceps weakness after reconstruction^15–18^. Furthermore, previous studies have found that greater preoperative muscular strength can improve outcomes^12,15^. Therefore, several studies have suggested adding preoperative exercise to increase quadriceps strength before ACL reconstruction surgery to achieve better functions after reconstruction^18,19^. However, we doubt whether this relationship is applicable in the setting of different grafts and different functional scores at different time periods for evaluation. Eitzen et al.^15^ evaluated knee functions in ACL reconstructed patients with bone-patellar -tendon-bone graft (BPTB) graft 2 years after surgery and Logerstedt et al.^16^ reported isometric muscle strength and functional score 6 months after ACL reconstruction. In addition, studies reported the mean time between surgery and resumption of sports of 7.3 (range 2–24) months^20^. Hence, 1 year may be appropriate to evaluate knee joint with the adequate recovery of muscle strength and functional ability for return to play.

Therefore, we evaluated isokinetic knee strength and subjective knee functions (Lysholm and IKDC scores) in participants with ACL reconstruction using hamstring graft 1 year after surgery. The primary aim of this study is to investigate the relationship between preoperative quadriceps strength and postoperative subjective knee functions after ACL reconstruction. The secondary aim is to see in which population the effect of quadriceps strength deficit on functional outcomes was most predominant.

## Methods

### Participants

This retrospective study enrolled all consecutive patients who underwent primary ACL reconstruction surgery between 2014 and 2017 in Samsung Medical Center. We included male patients with primary ACL reconstruction surgery who were preoperative evaluated and followed for more than 1 year. We excluded patients with a revision ACL reconstruction surgery, multi-ligamentous knee injury, prior ipsilateral lower limb surgeries, inflammatory joint disease, or other conditions affecting the lower limbs. All participants provided informed consent before participation in this study. The study was approved by the Sungkyunkwan University School of Medicine Clinical Research Ethics Board (SMC 2016-09-074-001). All methods were carried out in accordance with relevant guidelines and regulations.

### Surgical technique: ACL reconstruction

An anatomical, single-bundle ACL reconstruction surgery with quadruple semitendinosus and gracilis tendon autografts was performed by the same senior surgeon for all patients included in this study.

### Rehabilitation

All patients received an appropriate, graduated exercise rehabilitation program, consisting of home-based rehabilitation and supervised checkups while visiting the hospital at 15 days, 1, 3, and 9 months after surgery. In the first postoperative week, patients received light-intensity isometric and range of motion (ROM) exercises. The degree of knee flexion was increased by 15° each week in the ROM exercise program. In the 4th week, the knee joint ROM reached 90° and was increased to 120° in the 6th week. The patients were assigned for partial weight-bearing at 4–6 weeks postoperatively, progressing to full weight-bearing. At between 6 and 12 weeks, subjects performed combined strength, endurance, and balance exercises without pain. Light running was permitted at 6 months. Directional running and sports-related training were allowed at 9 months after surgery, in the absence of any swelling, pain, or knee instability.

### Muscle strength evaluation

A bioimpedance analyzer (InBody 170, BIOSPACE, KOREA) was used to measure the height, weight, body composition. It provides separate body mass readings for different segments of the body and uses an algorithm incorporating impedance, age and height in order to estimate total and regional body fat and fat-free mass. We evaluated quadriceps and hamstring muscle strength by the isokinetic dynamometer (CSMI Medical Solutions, Stoughton, MA, USA). After warm-up using a stationary bike for 10 min, the patients sit on the dynamometer and practiced the test protocol to become familiarized. The knee joint was moved 0°–90° at a speed of 60°/s (Nm) for isokinetic strength measurement. Finally, the patients were verbally encouraged to exert maximum effort on the machine. The patients performed four maximum repetitions of knee flexion and extension at 60°/s (Nm), and a 1 min resting period was allowed between consecutive tests. We recorded the hamstring quadriceps peak torque strength, quadriceps peak torque strength, and other indicators. The peak torque refers to the maximum acting torque released by the muscle during contraction. It is the torque value at the highest point of the torque curve and represents the maximum muscle force produced by muscle contraction. The mean peak torque of four repetitions for each velocity was calculated and compared with that on the uninjured side and described as absolute values and the percent of strength deficit calculated with the following formula:

$$ Strength~\,deficit = \left( {{\raise0.7ex\hbox{${noninjured\, limb - injured\,limb}$} \!\mathord{\left/ {\vphantom {{noninjured\,limb - injured\,limb} {noninjured\,limb}}}\right.\kern-\nulldelimiterspace} \!\lower0.7ex\hbox{${noninjured\,limb}$}}} \right) \times 100\%  $$

All isokinetic strength measurements were performed by the same experienced clinical physiologist, who was blinded to the testing result.

### Patient-reported outcome measures

The IKDC^21,22^ and Lysholm scores^23^ were used to measure subjective knee function. The IKDC score is a joint-specific outcome measure for assessing symptoms, function, and sports activity pertinent to various knee conditions^21^. The IKDC contains items regarding symptoms and disabilities important to patients with ACL deficiency^22^. The Lysholm score, a validated questionnaire, is used to evaluate and quantify patient satisfaction using 100 points scale that measures instability, limping, orthosis utilization, stair climbing, squatting, walking, running, jumping in daily life, pain, swelling, and thigh muscle atrophy^23^. The IKDC and Lysholm score forms are two widely used questionnaires among researchers and clinicians capable of measuring patient-reported knee function outcomes with good reliability and validity^14,24^.

### Follow-up

We assessed each participant’s knee function, including isokinetic muscle strength and functional score at baseline (preoperative) and at 12 months after index surgery.

### Statistical analysis

A priori calculated power > 0.80 at an alpha level equal to 0.05 was used to determine that a sample size of 45 for each group was necessary to determine R^2^. In this study, descriptive statistics were used to describe the basic features of the data. All the values are presented as median with interquartile range (IQR). The statistical analysis to determine associations between the two variables (preoperative quadriceps muscle strength deficit and the knee functional scores at 1 year after surgery) was conducted by the Spearman rank correlation analysis. We divided the patients into three groups according to the quadriceps muscle strength deficit. Although previous study used a 20% cut-off level for assessing the muscle strength deficit^15^, we set the muscle strength deficit 25% rather than 20% considering patients with unreconstructed ACL insufficiency knee may have a poor strength than those with ACL reconstructed knee. Meanwhile, ACL reconstructed patients could have a quadriceps strength deficit more than 45% during follow-up^12^ Therefore, the three identified groups (Q1–Q3) were classified according to the preoperative quadriceps muscle strength deficit differences. Group Q1 had the smallest difference with < 25% deficit in quadriceps muscle strength, Q2 showed a 25–45% deficit, and Q3 included patients who had > 45% deficit. Kruskal–Wallis test was used to compare parameters among three groups and Dunn test was used for post hoc analysis. Wilcoxon signed-rank test was used to compare baseline and postoperative functions within each group. A partial correlation analysis was then performed to determine the relationship between the knee functions at 1 year after surgery and the quadriceps muscle strength deficit in all patients and by level of strength deficit. Spearman’s partial correlation analysis, a nonparametric method, was used since some variables did not satisfy normality assumptions. Age, height, weight, and body fat percentage may affect the quadriceps muscle strength and were adjusted. The significance level was set at 0.05. All statistical analyses were performed using SPSS version 13.0 (SPSS Inc., Chicago, IL, USA) and MedCalc for Windows, version 18.6 (MedCalc Software, Ostend, Belgium).

## Results

Seventy-five male patients who underwent primary ACL reconstruction surgery were included in this study. The mean age was 32 (IQR, 26.3–39.8). Forty-seven (62.7%) had concomitant meniscus injury. Thirty-four patients received meniscus repair, and the remaining thirteen patients had partial meniscectomy. The patients were further divided into three groups according to the differences in preoperative quadriceps muscle strength deficit. Group Q1 (n = 26) had the smallest difference with < 25% deficit in quadriceps muscle strength, Q2 (n = 29) showed a 25–45% deficit, and Q3 included patients with a > 45% deficit (n = 20). There were no statistically significant differences in base demographics and the type of meniscus surgery between the three groups.

The baseline knee functional scores differed between the three groups, including IKDC score (*p* < 0.001) and Lysholm score (*p* < 0.001). There were also significant differences between the groups in knee extensor (*p* < 0.001) and flexor strength (*p* < 0.001). The baseline characteristics of the patients are shown in Table 1. At 1 year after reconstruction, the isokinetic strength and knee functional scores had improved (Supplemental Table 1). Q1 had significantly better isokinetic strength and knee function scores, including IKDC and Lysholm scores than Q3 (Table 2). Compared with Q2, Q1 had a trend of higher knee functions and isokinetic muscle strength, though not reaching the statistical significance.

**Table 1:** Characteristics of the study participants at baseline.

	Q1 (N = 26)	Q2 (N = 29)	Q3 (N = 20)	*p* value
**Demographics**				
Age (years)	31.5 (24.0–37.0)	33.0 (26.8–40.3)	32.0 (27.0–40.5)	0.668
Height (cm)	170.9 (167.6–175.6)	174.7 (170.0–178.0)	173.5 (167.5–176.0)	0.300
Weight (kg)	72.8 (68.8–80.4)	75.4 (68.4–79.7)	76.9 (72.0–79.5)	0.762
BMI (kg/m^2^)	25.3 (23.6–26.8)	24.5 (21.5–27.2)	24.7 (23.7–27.4)	0.665
Body fat (%)	22.9 (21.7–25.2)	23.7 (20.8–25.2)	23.6 (21.8–27.0)	0.621
**Meniscus treatment (n)**				0.993
Meniscal repair	11	13	10	
Meniscectomy	4	5	4	
**Baseline assessment**				
Subjective knee functional scores				
Subjective IKDC	78.3 (74.7–82.0)	72.5 (64.0–77.2)	59.8 (54.5–65.5)	< 0.001
Lysholm	80.0 (72.0–82.0)	75.0 (65.0–77.5)	63.5 (61.0–69.5)	< 0.001
Muscle strength (Nm, at 60°/s)				
Quadriceps	96.5 (90.0–109.0)	82.0 (71.0–94.0)	58.5 (54.5–68.0)	< 0.001
Hamstring	69.0 (56.0–77.0)	59.0 (45.8–67.3)	45.5 (41.0–59.0)	< 0.001
Muscle strength deficit (%)				
Quadriceps	20.9 (14.4–23.3)	33.9 (29.3–39.7)	48.6 (46.5–51.0)	< 0.001
Hamstring	13.5 (10.0–16.9)	22.8 (10.0–29.3)	31.8 (21.0–36.1)	< 0.001

Group Q1: < 25% deficit in quadriceps muscle strength, group Q2: 25–45% deficit quadriceps muscle strength, and group Q3: > 45% deficit in quadriceps muscle strength, compared the uninjured limb.

*BMI* body mass index, *IKDC* International knee documentation committee.

Values are presented as median [IQR 25–75th percentile] if no otherwise specified.

**Table 2:** Knee functions at 1 year after surgery.

	Q1 (N = 26)	Q2 (N = 29)	Q3 (N = 20)	*p* value
**Assessment at 1 year**				
Subjective knee functional scores				
Subjective IKDC	85.2 (80.0–90.8)	81.2 (77.1–85.1)	80.3 (76.5–83.0)^a^	0.009
Lysholm	95.0 (92.0–99.0)	90.0 (85.0–95.0)	89.5 (81.5–90.5)^a^	0.001
Muscle strength (Nm, at 60°/s)				
Quadriceps	105.0 (100.0–116.0)	93.0 (80.5–115.5)	91.5 (75.0–105.0)^a^	0.047
Hamstring	68.5 (64.0–84.0)	69.0 (53.5–80.3)	60.0 (46.5–70.0)^a^	0.020
Muscle strength deficit (%)				
Quadriceps	15.3 (11.6–18.0)	21.9 (14.7–31.2)^a^	21.7 (15.7–28.7)^a^	0.002
Hamstring	9.5 (4.8–15.8)	9.9 (7.8–19.1)	16.1 (5.1–23.8)	0.196

Group Q1: < 25% deficit in quadriceps muscle strength, group Q2: 25–45% deficit quadriceps muscle strength, and group Q3: > 45% deficit in quadriceps muscle strength, compared the uninjured limb.

*IKDC* International Knee Documentation Committee.

Values are presented as median [IQR 25–75th percentile] if not otherwise specified.

^a^*p* < 0.05 by post hoc Dunn test, compared with Q1.

### Relationship between preoperative quadriceps muscle strength and postoperative knee functional score

**Figure 1:**
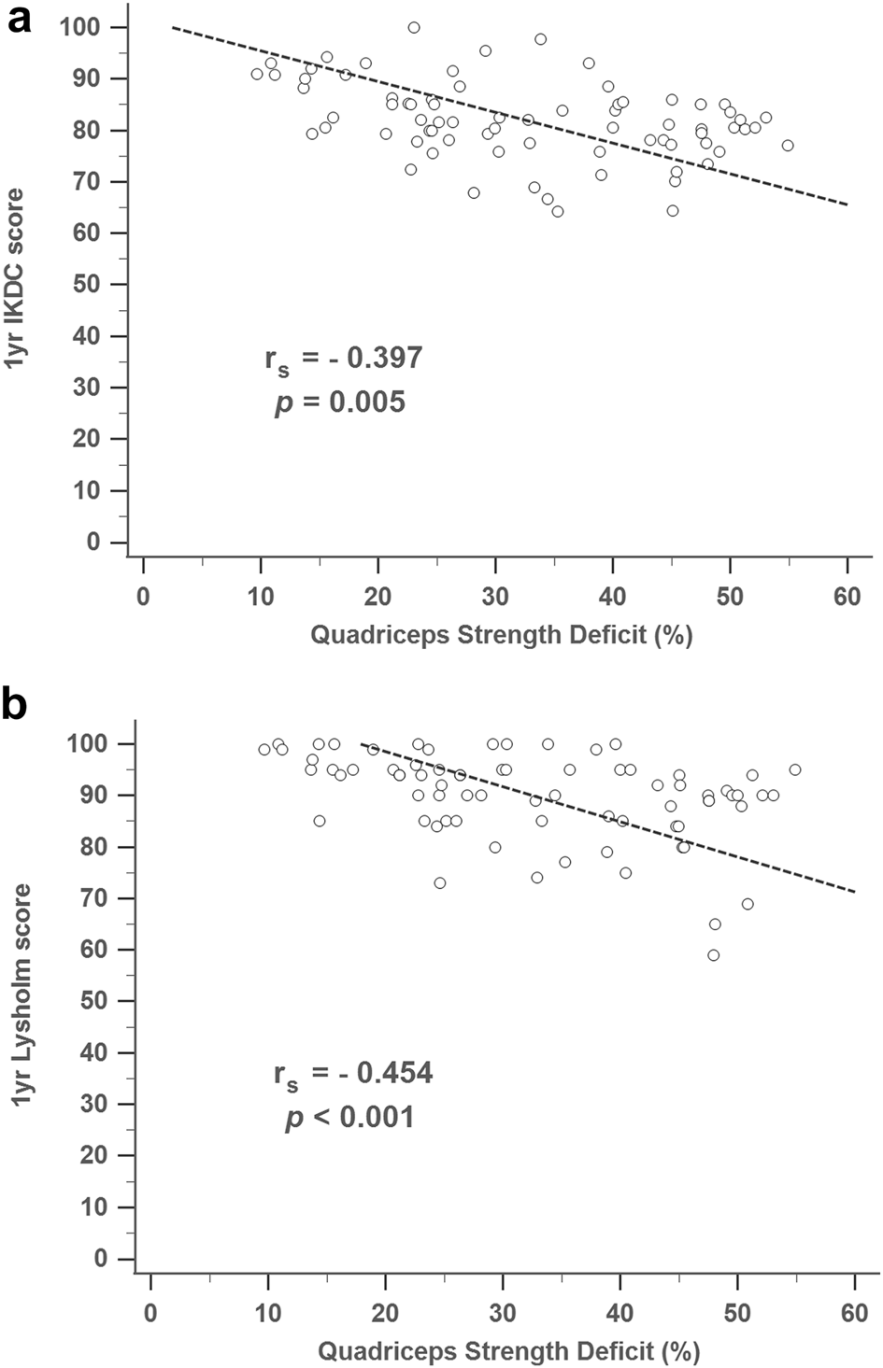
Association between knee extension strength deficit and subjective knee functional score at 1 year. (**a**) IKDC score, IKDC, International Knee Documentation Committee; (**b**) Lysholm score.

The preoperative quadriceps muscle strength deficit had a negative association with the knee functional scores at 1 year follow-up including the IKDC score (r_s_ =−0.397, *p* = 0.005, Fig. 1a) and the Lysholm score (r_s_ = − 0.454, *p* < 0.001, Fig. 1b).

### Postoperative knee functional score by levels of quadriceps muscle strength deficit

**Figure 2:**
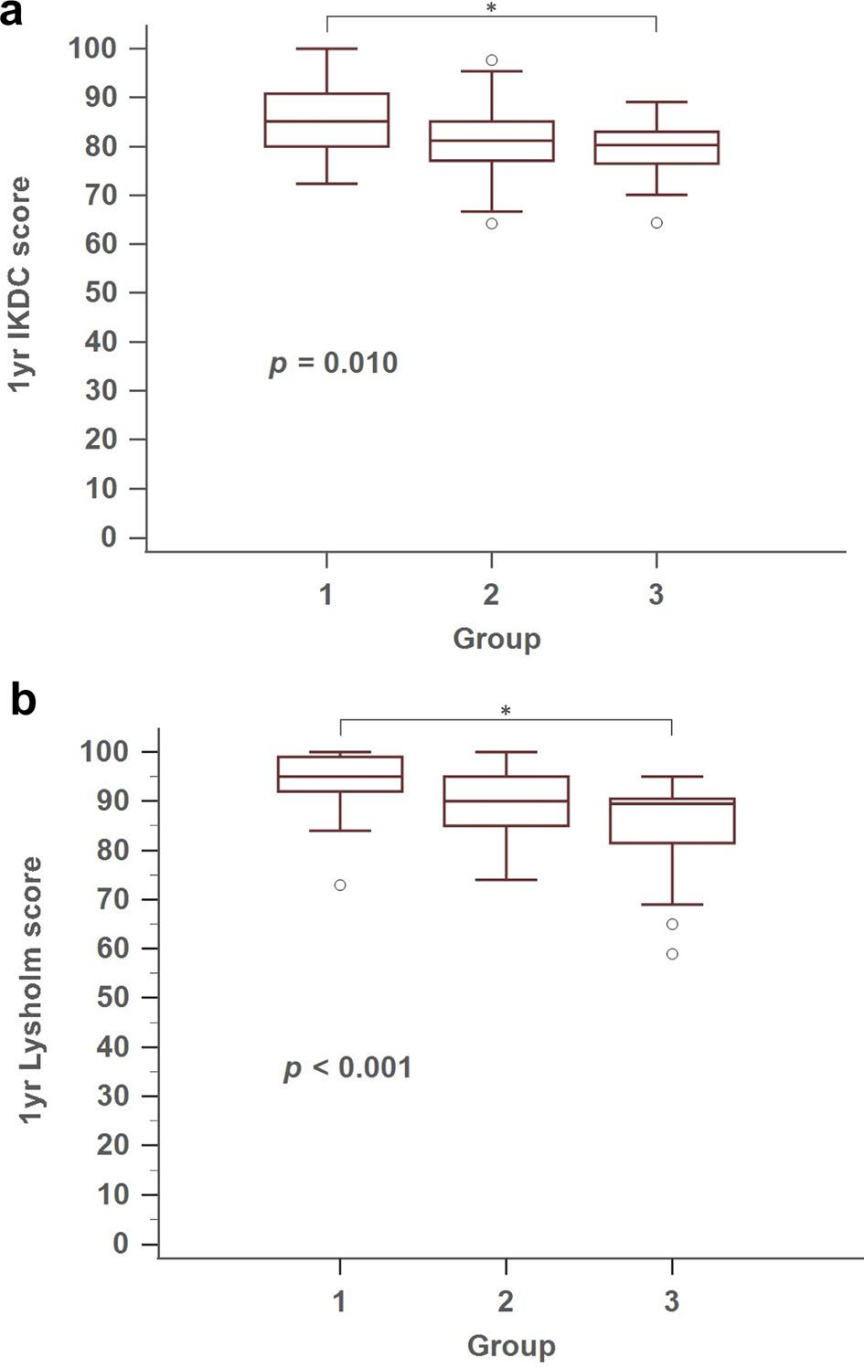
Comparison of subjesctive knee functional score at 1 year, between groups. (**a**) IKDC score, IKDC, International Knee Documentation Committee; (**b**) Lysholm score. Asterisks indicate statistically significant differences between the two measurements by post hoc Dunn test (*p* < 0.05).

When comparing knee function at 1 year after surgery, the three groups were significantly different in knee functional score, including IKDC (*p* = 0.010) and Lysholm score (*p* < 0.001) (Fig. 2a,b).

Partial correlation analysis was performed to examine the correlation between knee functional scores by different preoperative quadriceps muscle strength deficit levels (Table 3). Only the Q1 group (< 25% difference in preoperative quadriceps muscle strength) showed a statistically significant correlation in postoperative knee functions, measured by the IKDC score and Lysholm score. The Q2 and Q3 groups, which had more than 25% difference in preoperative quadriceps muscle strength, showed no significant association with knee functional scores 1 year after surgery, neither by IKDC nor Lysholm scores. In addition, preoperative quadriceps muscle strength deficit level had a statistically significant negative correlation with subjective knee functions including IKDC score and Lysholm score (Supplemental Table 2).

**Table 3:** Summary of partial correlation coefficients for subjective knee function at 1 year with preoperative knee extension strength deficit across groups.

	Q1 (< 25% deficit) (n = 26)		Q2 (25–45% deficit) (n = 29)		Q3 (> 45% deficit) (n = 20)	
	Correlation coefficient	*p* value	Correlation coefficient	*p* value	Correlation coefficient	*p* value
IKDC score	− 0.462	0.030*	− 0.072	0.732	0.482	0.059
Lysholm score	− 0.446	0.038*	− 0.139	0.507	− 0.215	0.424

Age, height, weight, and body fat percentage were adjusted.

Values are presented as median [IQR 25–75th percentile] if no otherwise specified.

*IKDC* international knee documentation committee.

*p < 0.05.

## Discussion

This study’s principal findings showed that preoperative quadriceps strength deficit negatively predicted knee functional scores 1 year after ACL reconstruction surgery. Patients with more significant preoperative quadriceps strength deficit would have poor knee functions even after rehabilitation. Therefore, preoperative rehabilitation, including quadriceps strengthening, may be crucial to functional recovery after ACL reconstruction surgery.

Muscle weakness in the lower limbs appears to be due mainly to neuromuscular dysfunction and decreased activity level after ACL injury^25,26^ and the restricted ROM during the recovery phase after ACL reconstruction. In particular, weakening of the quadriceps muscles is known to negatively affect knee functions after reconstruction^11,27,28^ The degree of strength deficit after ACL reconstruction is one of the important indicators when evaluating the state of the knee joint, and clinically no more than a 10% difference should be permitted for patients returning to sport^3,29^. However, studies conducted on the extent of strength deficit after ACL reconstruction have found quadriceps muscle strength deficits of 10–27% after 1 year, and the deficiency remained at 6–10% even after more than 5 years following surgery^30–32^.

Preoperative quadriceps strength is positively associated with postoperative functional outcomes in available evidence^12,15,16,33,34^. Both postoperative single leg hoop distance index (HDI)^12,33^ and patient-reported outcomes, including IKDC score^16^, Cincinnati Knee Scale^15^ and KOOS-QOL^34^, had a positive association with preoperative quadriceps strength^35,36^. The current study showed that the IKDC and Lysholm scores have a significant association with preoperative quadriceps muscle strength. These results were consistent with the findings of Eitzen et al.^15^, in that the preoperative quadriceps muscle strength can be used to predict 15.6% of the variance in the Cincinnati knee score 2 years after ACL reconstruction with bone-patellar-tendon-bone graft. Preoperative quadriceps strength correlated significantly with 6 months postoperative HDI^12,33^. Furthermore, De Jong et al.^12^ found a statistically significant difference in HDI at both 6 and 9 months after surgery if the preoperative deficit in quadriceps muscle strength was greater than 20%. In the current study, we further divided patients into three groups according to the quadriceps strength deficit's extent and measured the association between each group and the selected outcome variables. The group (Q3) with preoperative quadriceps strength deficit greater than 45% had a significant poor knee function measured by IKDC and Lysholm scores and isokinetic extensor/flexor strengths 1 year after surgery than the Q1 group (preoperative muscle strength deficit less than 25%). Furthermore, a significant negative correlation between quadriceps strength deficit and knee function 1 year after surgery was also noted in the Q1 group. That is, preoperative muscle deficit was crucial for postoperative knee functions and muscle strengths.

In addition to preoperative quadriceps strength, several factors were associated with subjective knee functions after ACL reconstruction^28,37,38^. Ueda et al.^28^ showed that the greater preoperative quadriceps muscle strength deficit affects not only the longitudinal postoperative quadriceps strength index recovery but also high-level 1-leg hop performance after ACL reconstruction using hamstring autograft. In addition, Balki et al.^37^ further indicated that hamstring weakness at 90° flexion of the involved knee is an indicator of the function deficit in males after ACL reconstruction. Maguire et al.^38^ showed that both male and female athletes demonstrated persistent severe hamstring strength deficits at 8 months after ACL reconstruction, contributing to poor subjective knee functions. In the current study, the Q2 and Q3 groups had similar quadriceps muscle deficits, and Q3 had an even greater hamstring deficit than Q2 1 year after surgery. However, our data failed to find a significant association between hamstring deficit and subjective knee functions 1 year after surgery due to the limitation of the sample size. A future study focused on the issue may be needed to solve the debate.

Based on the results of previous studies and this study, weakness of the quadriceps muscle after ACL reconstruction is apparent, and it is tough to recover the strength of the quadriceps muscle even after months or years following reconstruction. Ultimately, this can affect the knee joint function after ACL reconstruction^15,30,32^. Therefore, various approaches aimed at preventing further weakening of the quadriceps muscle strength following ACL injury and surgery should be considered. The recent systematic review supported the effectiveness of preoperative training on postoperative knee functions after ACL reconstruction^39^. Our previous study also confirmed the positive effects of 4 weeks preoperative exercise on knee extensor strength after ACL reconstruction^19^. However, in reality, most patients do not start physical therapy before ACL reconstruction surgery, which likely adds to preventable muscle weakening. Therefore, medical professionals should be encouraged to be aware of physical therapy's importance before ACL surgery and then plan safe, systematic physical therapy programs for patients.

Our study had several limitations. First, we did not have priori sample calculation and included a limited number of male patients with primary ACL reconstruction surgery in this study. The conclusion may be changed or reach the statistical significance after enrolling a larger sample, especially in each subgroup. Additionally, external validity of our study is limited, especially for female patients or revision ACL surgeries. Further studies on females may be needed to clarify these findings. Second, we cannot address whether the meniscus injury pattern impacts the outcomes since the severity of meniscus injury differed among patients. Since meniscus repair did not alter limb symmetry recovery^40^, and the treatments for meniscus injury were not significantly different across the three groups in the current study, this potential bias should be ignored. Third, we do not know whether the difference of knee function reaches minimal clinically important significance since we did not record the status of return to play. Fourth, we did not take leg dominance into consideration in the current study. The influence of leg dominance on muscle strength and return to sports after ACL reconstruction remained uncertain^41,42^. Suh et al.^41^ showed the quadriceps strength of the operated non-dominant leg was lower than that of the operated dominant leg 6 months postoperatively; however, the strength of the quadriceps and hamstring muscles was not different after 12 months between the operated dominant and non-dominant legs. Since the current study evaluated the knee functions 1 year after ACL reconstruction, the influence of leg dominance might be ignored. Lastly, we did not record the pre-injury level of physical activity as mentioned earlier. Therefore, we have no idea the influence of pre-injury level of activity on the outcomes following ACL reconstruction surgery based on the current study. A future study recording the pre-injury level of physical activity is needed to solve this question.

## Conclusions

This study showed that preoperative quadriceps muscle strength deficit had a significant negative relationship with postoperative function at 1 year following ACL reconstruction. Meanwhile, patients with more significant preoperative quadriceps strength deficit would have poor knee functions than those with better preoperative quadriceps strength, even after rehabilitation. Therefore, restoring muscle strength by preoperative exercise may be crucial for a better function after ACL reconstruction surgery.

## Supplementary Information

Supplementary Tables.
