# Supplementary material for: Preoperative quadriceps muscle strength deficit severity predicts knee function one year after anterior cruciate ligament reconstruction
Source: Sci Rep. 2022 Apr 6;12:5830. doi: 10.1038/s41598-022-09816-3 (PMC8986796; doi:10.1038/s41598-022-09816-3)
Supplement: Supplementary file 1 — Supplementary Tables. [file 41598_2022_9816_MOESM1_ESM.docx]

**Supplemental Table 1.** Comparisons of knee functions between baseline and one year after surgery

|  | Q1  (N=26) | *p* value | Q2  (N=29) | *p* value | Q3  (N=20) | *p* value |
| --- | --- | --- | --- | --- | --- | --- |
| Subjective knee functional scores |  |  |  |  |  |  |
| Subjective IKDC | 78.3 (74.7 – 82.0) | <0.001 | 72.5 (64.0 – 77.2) | 0.001 | 59.8 (54.5 – 65.5) | <0.001 |
|  | 85.2 (80.0 – 90.8) |  | 81.2 (77.1 – 85.1) |  | 80.3 (76.5 – 83.0) |  |
| Lysholm | 80.0 (72.0 – 82.0) | <0.001 | 75.0 (65.0 – 77.5) | <0.001 | 63.5 (61.0 – 69.5) | <0.001 |
|  | 95.0 (92.0 – 99.0) |  | 90.0 (85.0 – 95.0) |  | 89.5 (81.5 – 90.5) |  |
| Muscle Strength  (Nm, at 60°/s) |  |  |  |  |  |  |
| Quadriceps | 96.5 (90.0 – 109.0) | 0.016 | 82.0 (71.0 – 94.0) | <0.001 | 58.5 (54.5 – 68.0) | <0.001 |
|  | 105.0 (100.0 – 116.0) |  | 93.0 (80.5 – 115.5) |  | 91.5 (75.0 – 105.0) |  |
| Hamstring | 69.0 (56.0 – 77.0) | 0.023 | 59.0 (45.8 – 67.3) | 0.001 | 45.5 (41.0 – 59.0) | 0.016 |
|  | 68.5 (64.0 – 84.0) |  | 69.0 (53.5 – 80.3) |  | 60.0 (46.5 – 70.0) |  |

Group Q1: <25% deficit in quadriceps muscle strength, group Q2: 25-45% deficit quadriceps muscle strength, and group Q3: > 45 % deficit in quadriceps muscle strength, compared the uninjured limb.

Abbreviations: *IKDC* International Knee Documentation Committee.

Values are presented as median [IQR, 25th-75th percentile] if not otherwise specified.

**Supplemental Table 2.** Partial Correlation Coefficients for subjective knee function at one year with preoperative knee extension strength deficit level^a^

| Outcome | Correlation coefficient | *p* value |
| --- | --- | --- |
| IKDC score | -0.3628 | 0.0019 |
| Lysholm score | -0.3510 | 0.0027 |

Abbreviation: IKDC, international knee documentation committee

^a^ Age, height, weight, and body fat percentage were adjusted.
